# Supplementary material for: Seminoma and Embryonal Carcinoma Footprints Identified by Analysis of Integrated Genome-Wide Epigenetic and Expression Profiles of Germ Cell Cancer Cell Lines
Source: PLoS One. 2014 Jun 2;9(6):e98330. doi: 10.1371/journal.pone.0098330 (PMC4041891; doi:10.1371/journal.pone.0098330)
Supplement: File S1 — ZIP file containing DMRforPairs output for significant regions. Please start from the html files. (ZIP) [file pone.0098330.s008.zip › figures/6084.pdf]

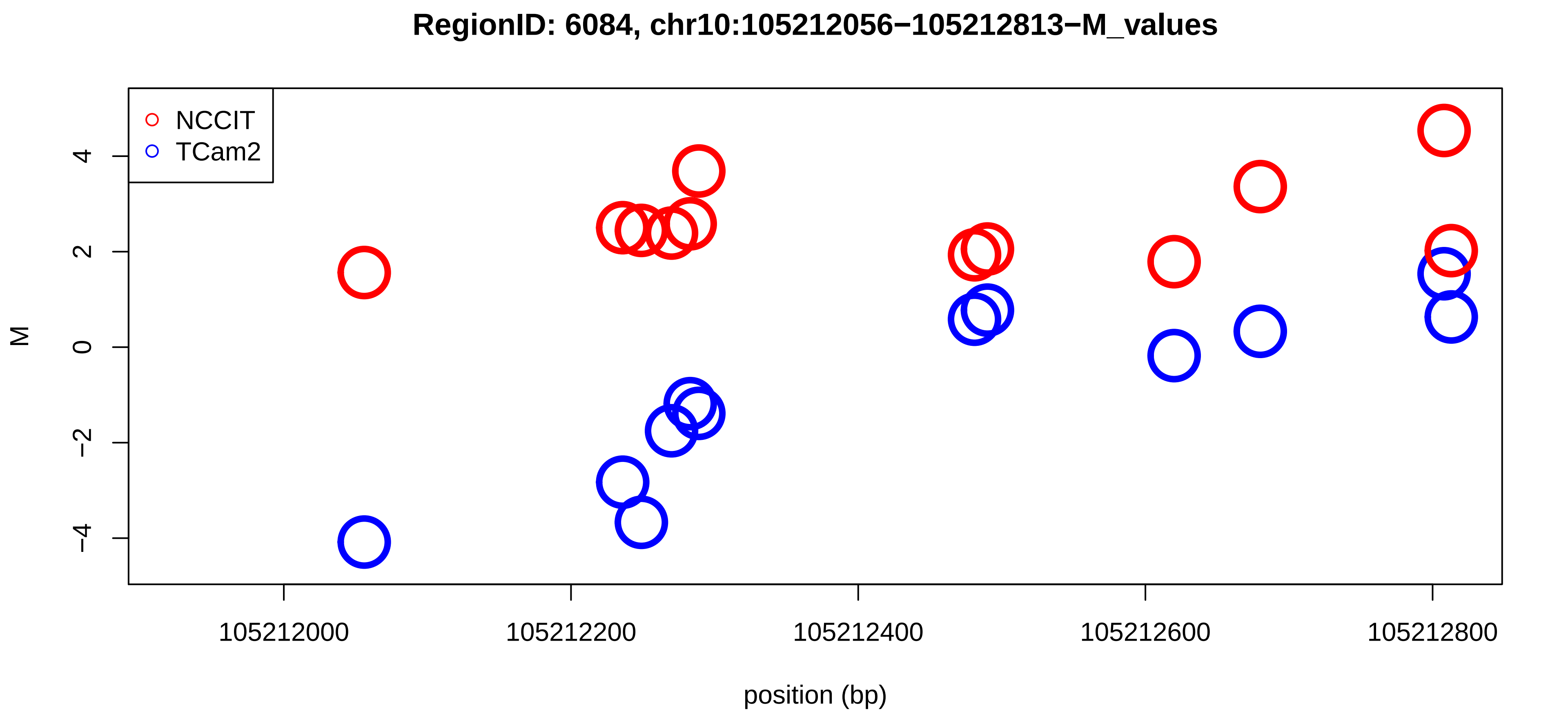
RegionID: 6084, chr10:105212056−105212813−M_values
NCCIT
TCam2
M
4
2
0
−2
−4
105212000
105212200
105212400
105212600
105212800
position (bp)

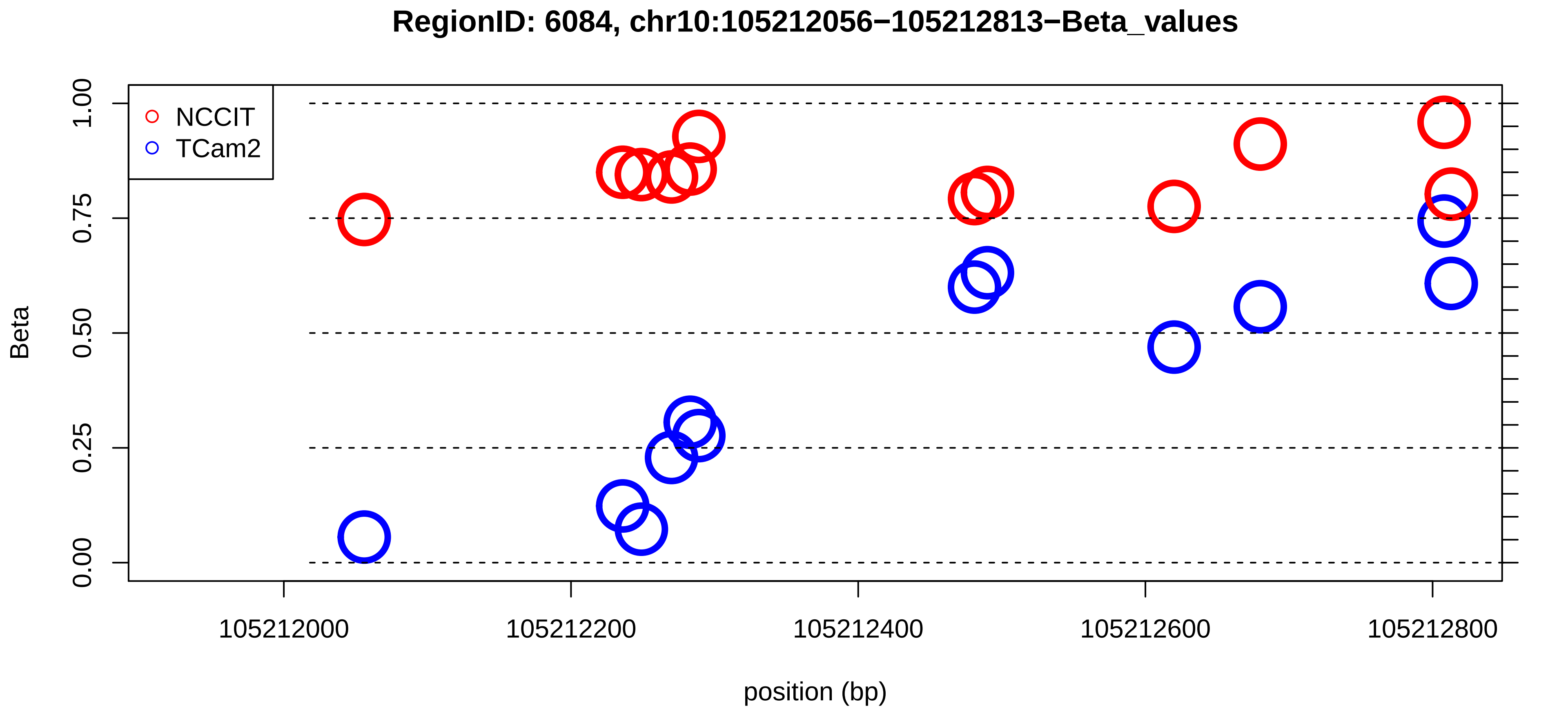
RegionID: 6084, chr10:105212056−105212813−Beta_values
NCCIT
TCam2
Beta
1.00
0.75
0.50
0.25
0.00
105212000
105212200
105212400
105212600
105212800
position (bp)

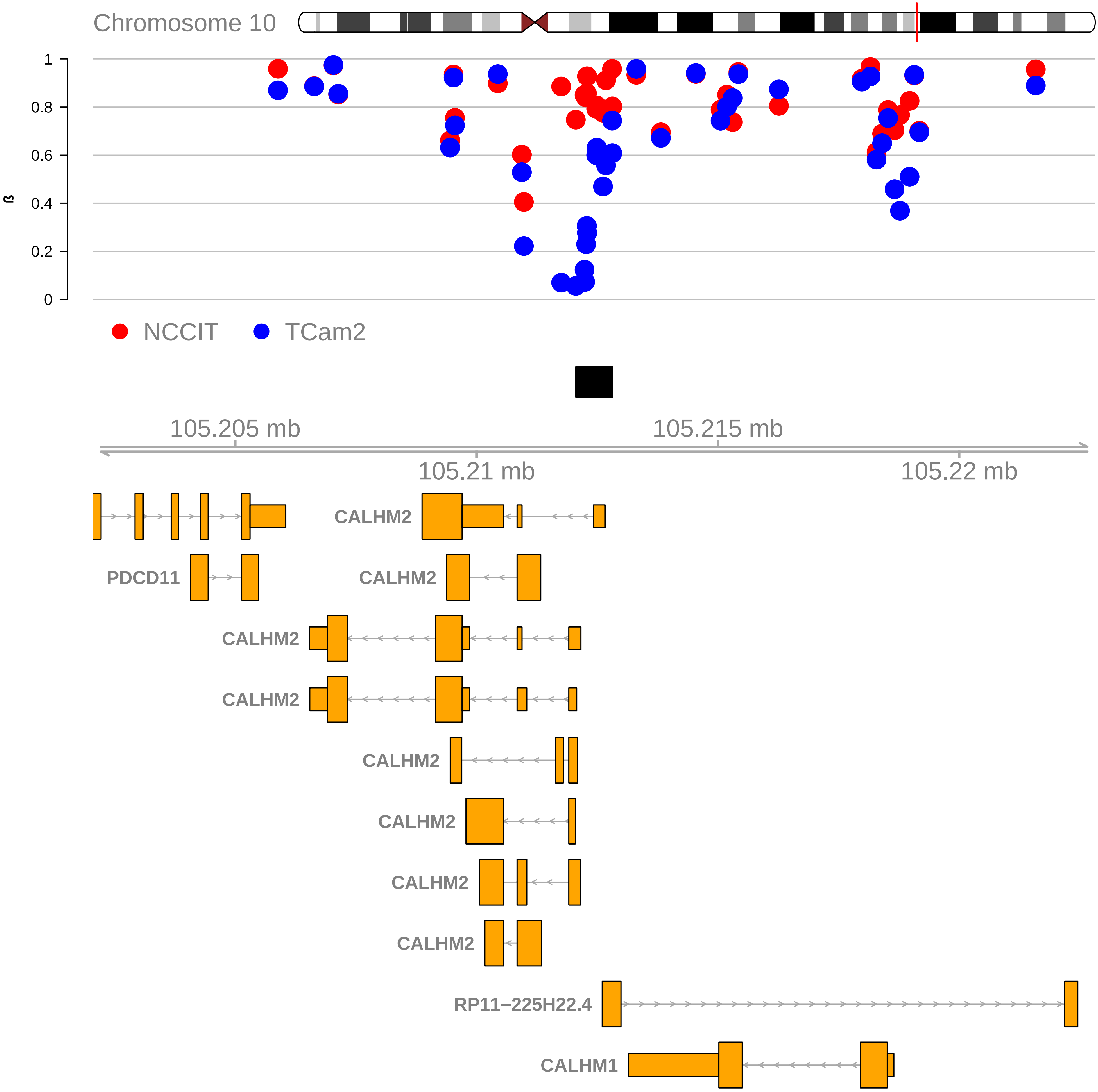
Chromosome 10
ß
1
0.8
0.6
0.4
0.2
0
NCCIT
TCam2
105.205 mb
105.215 mb
105.21 mb
105.22 mb
CALHM2
PDCD11
CALHM2
CALHM2
CALHM2
CALHM2
CALHM2
CALHM2
CALHM2
RP11−225H22.4
CALHM1
